# Dependence of the yields of AP sites and AP clusters produced in plasmid DNA on scavenging capacity and LET

**DOI:** 10.1093/jrr/rrt160

**Published:** 2014-03

**Authors:** Takuya Shiina, Ritsuko Watanabe, Masao Suzuki, Akinari Yokoya

**Affiliations:** 1 Ibaraki University, Japan; 2 JAEA, Japan; 3 NIRS, Japan

**Keywords:** AP site, track structure, Monte Carlo simulation, carbon ion beam

## Abstract

An apurinic or apyrimidinic site (AP site) has known to be one of the typical DNA lesions induced by ionizing irradiation to cells. A clustered DNA damage site composed of AP sites (AP clusters) can be visualized as a double-strand break (DSB) by a treatment of DNA sample with endonuclease IV proteins as an enzymatic probe. AP clusters are efficiently induced in human cells by low LET γ- and X-irradiation [ 1] with the similar yields with those for the clusters which contain pyrimidine or purine base lesions. However, there has been very little knowledge of the mechanistic aspects of the production of AP sites and AP clusters. Recently, we reported the dependence of the yields of AP sites and AP clusters, as well as base lesions, strand breaks and base lesion clusters induced by irradiation of carbon ions (LET: 13 and 60 keV/μm) obtained from HIMAC (NIRS, Chiba, Japan) on radical scavenging capacity in the DNA samples [ 2].

In the present study, we have performed theoretical calculation for the production of AP sites as well as those of strand breaks and base lesions by the carbon ion exposure (13 keV/μm). A Monte Carlo track structure simulation code for ion tracks, TRACION, was used for this work according to our previous study [ 3]. As a DNA model molecule, simple linear DNA with 150 bp was applied for the calculation. Several scavenging capacities of the samples are tested to estimate the effect of indirect action of diffusible OH radicals in the damage induction in the experimental conditions.

We assumed that the AP site formation pathway originates from a common intermediate of an OH radical adduct with that of single-strand breaks (SSBs) or base lesions (Scheme 1). In order to reproduce the experimentally obtained yields of AP sites, we newly determined the branching ratio of induction of AP site to that of SSBs or base lesions to be 17:33 or 2:48, respectively, in the simulation. The calculated yields of AP site, as well as SSB, were well consistent with those for experimentally obtained (Fig. 1). However, the calculated yields for AP clusters were one-fifth of those for experimental data. The yields for DSBs in lower scavenging capacities were also one-fifth of those for experimental ones in a lower scavenging capacity, thought they were well consistent with each other above a cell mimetic condition (>3 × 10^8^ s). In order to bridge a gap between theoretical and experimental yields, we need a novel mechanism of damage-clustering such as dissociative low energy electron attachment or hole-migration onto a DNA molecule.

**Fig. 1.**
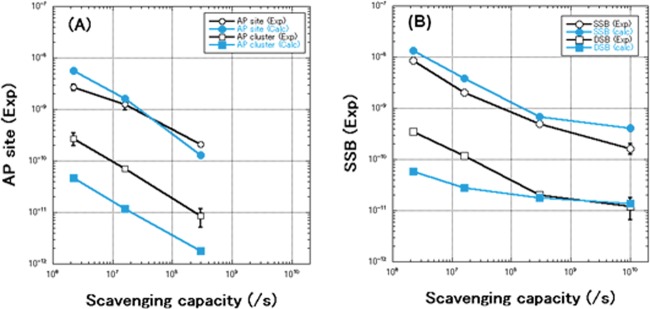
Dependence of the yield of AP site or AP cluster (A) and prompt SSB or prompt DSB (B) on scavenging capacity in the sample. The experimental data below a scavenging capacity of 10^9^ s are cited from [ 2] and those at 10^10^ s in (B) are from [ 4].

**Scheme 1.**
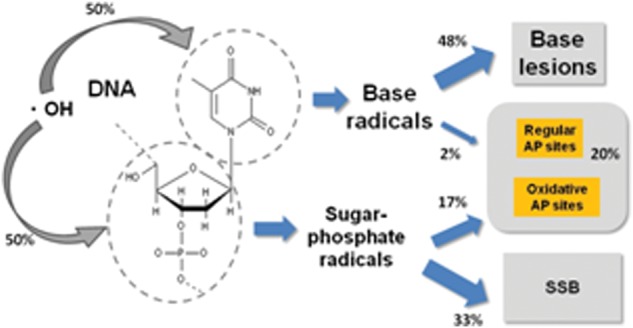
Reactions of DNA with an OH radical and the branching ratios among the damage induction pathways.

Dependence of the yield of AP site or AP cluster (A) and prompt SSB or prompt DSB (B) on scavenging capacity in the sample. The experimental data below a scavenging capacity of 10^9^ s are cited from [ 2] and those at 10^10^ s in (B) are from [ 4].

Reactions of DNA with an OH radical and the branching ratios among the damage induction pathways.
